# Predictive Value of T_**peak**_ – T_**end**_ Indices for Adverse Outcomes in Acquired QT Prolongation: A Meta-Analysis

**DOI:** 10.3389/fphys.2018.01226

**Published:** 2018-09-03

**Authors:** Gary Tse, Mengqi Gong, Lei Meng, Cheuk W. Wong, George Bazoukis, Matthew T. V. Chan, Martin C. S. Wong, Konstantinos P. Letsas, Adrian Baranchuk, Gan-Xin Yan, Tong Liu, William K. K. Wu

**Affiliations:** ^1^Department of Medicine and Therapeutics, Faculty of Medicine, Chinese University of Hong Kong, Hong Kong, Hong Kong; ^2^Li Ka Shing Institute of Health Sciences, Faculty of Medicine, Chinese University of Hong Kong, Hong Kong, Hong Kong; ^3^Tianjin Key Laboratory of Ionic-Molecular Function of Cardiovascular Disease, Department of Cardiology, Tianjin Institute of Cardiology, Second Hospital of Tianjin Medical University, Tianjin, Hong Kong; ^4^Li Ka Shing Faculty of Medicine, University of Hong Kong, Hong Kong, Hong Kong; ^5^Laboratory of Cardiac Electrophysiology, Second Department of Cardiology, Evangelismos General Hospital of Athens, Athens, Greece; ^6^Department of Anaesthesia and Intensive Care, Chinese University of Hong Kong, Hong Kong, Hong Kong; ^7^JC School of Public Health and Primary Care, Chinese University of Hong Kong, Hong Kong, Hong Kong; ^8^Division of Cardiology, Kingston General Hospital, Queen's University, Kingston, ON, Canada; ^9^Lankenau Institute for Medical Research and Lankenau Medical Center, Wynnewood, PA, United States; ^10^Beijing Anzhen Hospital, Capital Medical University, Beijing, China

**Keywords:** T_**peak**_—T_**end**_, T_**peak**_—T_**end**_/QT, dispersion of repolarization, risk stratification, ventricular arrhythmia, sudden cardiac death

## Abstract

**Background:** Acquired QT interval prolongation has been linked with malignant ventricular arrhythmias, such as *torsade de pointes*, in turn predisposing to sudden cardiac death. Increased dispersion of repolarization has been identified as a pro-arrhythmic factor and can be observed as longer T_peak_ – T_end_ interval and higher T_peak_ – T_end_/QT ratio on the electrocardiogram. However, the values of these repolarization indices for predicting adverse outcomes in this context have not been systematically evaluated.

**Method:** PubMed, Embase and Cochrane Library databases were searched until 14th February 2018, identifying 232 studies.

**Results:** Five studies on acquired QT prolongation met the inclusion criteria and 308 subjects with drug-induced LQTS patients (mean age: 66 ± 18 years old; 46% male) were included in this meta-analysis. T_peak_ – T_end_ intervals were longer [mean difference [MD]: 76 ms, standard error [SE]: 26 ms, *P* = 0.003; I^2^ = 98%] and T_peak_ – T_end_/QT ratios were higher (MD: 0.14, SE: 0.03, *P* = 0.000; I^2^ = 29%) in patients with *torsade de pointes* compared to those without these events.

**Conclusion:** T_peak_ – T_end_ interval and T_peak_ – T_end_/QT ratio were higher in patients with acquired QT prolongation suffering from *torsade de pointes* compared to those who did not. These repolarization indices may provide additional predictive value for identifying high-risk individuals.

## Introduction

Acquired prolongation of QT interval increases the risk of ventricular tachycardia/fibrillation (VT/VF) and sudden cardiac death (SCD). However, patients with prolonged QT intervals are heterogeneous in that only a subset develop these adverse events and risk stratification remains difficult. Of the different electrophysiological mechanisms, exacerbated repolarization gradient across the myocardial wall has been identified as an important factor of arrhythmogenesis in the context of QT prolongation, as it can lead to unidirectional block and reentrant activity (Opthof et al., 2007; Coronel et al., 2009; Choy et al., 2016). On the electrocardiogram, the dispersion of repolarization can be estimated using the T_peak_ – T_end_ Interval, defined as the time difference between the peak and the end of the T-wave. However, this parameter varies with heart rate (Gupta et al., 2008) and normalization with QT interval (T_peak_ – T_end_/QT ratio) gives a relatively constant range of values between 0.17 and 0.23 (Gupta et al., 2008). Despite their strong physiological basis, the predictive value of both parameters in drug-induced QT prolongation remains controversial.

A recent meta-analysis reported that higher T_peak_ – T_end_ interval was predictive of adverse events that include ventricular tachyarrhythmias and sudden cardiac death in congenital long QT syndrome, but it did not investigate its value in cases of acquired QT interval prolongation (Tse et al., 2017a). Nevertheless, several reports have investigated the differences in T_peak_ – T_end_ interval and/or T_peak_ – T_end_ / QT ratio between event-positive and event-negative subjects with acquired QT prolongation with some conflicting results. In this study, we performed a systematic review and meta-analysis to elucidate the relationship between these parameters and adverse events in this patient population.

## Methods

### Search strategy, inclusion, and exclusion criteria

This systematic review and meta-analysis was performed according to the Preferred Reporting Items for Systematic Reviews and Meta-Analyses (PRISM) statement (Moher et al., 2015). PubMed, Embase and Cochrane Library were searched for studies that investigated the association between T_peak_ – T_end_ or T_peak_ – T_end_/QT with arrhythmic or mortality endpoints in long QT syndrome. The following search terms were used for all three databases: [“Tpeak–Tend” OR “Tp-Te” OR “Tpeak-end” OR “Tp-e” OR “T(peak)-T(end)” OR “T wave peak-to-end” OR “T peak-T end” OR “Tpe” “TPEc” OR “T-peak to T-end” OR “Tpeak-to-Tend” AND “long QT”]. The search period was from the beginning of the database through to 14th February 2018 without language restrictions. The following inclusion criteria were used: (i) the study was conducted in humans, (ii) T_peak_ – T_end_ intervals or T_peak_ – T_end_/QT ratios between event-positive (ventricular tachycardia/fibrillation, or sudden cardiac death) and event-negative groups were compared.

The quality assessment of these studies included in our meta-analysis was performed using the Newcastle–Ottawa Quality Assessment Scale (NOS; Marshall et al., 2007). The details of the NOS quality assessment are shown in Supplementary Tables 1, 2. No studies were excluded because of the quality score.

### Data extraction and statistical analysis

Data from the different studies were entered in pre-specified spreadsheets in Microsoft Excel (Version 2016). All search entries were retrieved as complete manuscripts and assessed to determine whether they met the inclusion criteria. The extracted data elements consisted of: (i) publication details: last name of first author, publication year; (ii) study design; (iii) endpoint(s); (iv) the quality score; and (v) the characteristics of the population including sample size, gender, age and number of subjects. Two reviewers (GT and MG) reviewed each included study independently. Disagreements were resolved by consulting with a third reviewer (TL).

Mean differences with 95% confidence interval (CI) for T_peak_ – T_end_ interval and T_peak_ – T_end_/QT ratio were extracted from each study and subsequently pooled. Heterogeneity between studies was determined using Cochran's *Q*-value, the weighted sum of squared differences between individual study effects and the pooled effect across studies, and *I*^2^ from the standard chi-square test, which describes the percentage of the variability in effect estimates resulting from heterogeneity. *I*^2^ > 50% was considered to reflect significant statistical heterogeneity. A fixed effects model was used if *I*^2^ < 50%, otherwise the random-effects model using the inverse variance heterogeneity method was used. To locate the origin of the heterogeneity, sensitivity analysis excluding one study at a time were performed. Funnel plots, Begg and Mazumdar rank correlation test and Egger's test were used to assess for possible publication bias.

## Results

A flow diagram of the search strategy is shown in Supplementary Figure 1. Our search retrieved 99 entries from PubMed, 116 entries from Embase and 17 from Cochrane Library. Of these, five studies were relevant for acquired LQTS and were included in our final meta-analysis (Yamaguchi et al., 2003; Topilski et al., 2007; Darbar et al., 2008; Couderc et al., 2010; Subbiah et al., 2010). A total of 308 patients were included (mean age: 66 ± 18 years old; 46% male). Table 1 shows the baseline characteristics of these studies and of the study populations.

**Table 1:** Characteristics of the five studies included in this meta-analysis.

**First author/Year**	**Study design**	**Population**	**Drugs**	**Lead for T_peak_–T_end_ measurement**	**Method of T_end_ measurement**	**Adverse events**	**Sample size (*n*)**	**Age**	**% Male**	**Endpoints**	**No. of patients with any positive events**	**References**
Couderc 2010	Retrospective	Drug-induced LQTS	Sotolol	All 12 leads	Tangent method	TdP	6	50	67	TdP	3	Couderc et al., 2010
Subbiah 2010	Prospective	AV block with TdP	–	All 12 leads	Tangent method	TdP	40	70	40	TdP	7	Subbiah et al., 2010
Darbar 2008	Retrospective	Drug-induced LQTS	Quinidine; sotalol, dofetilide, procainamide, disopyramide, amiodarone, trimethoprim-sulfamethoxazole, phenothiazines, Haloperidol, Fluconazole /itraconazole, cisapride, pentamidine, lithium, terfernadine, clarithromycin	All 12 leads	Baseline method	TdP	123	59	20	TdP	83	Darbar et al., 2008
Topilski 2007	Retrospective	AV block with TdP	–	All 12 leads	Baseline method	TdP	143	76	19	TdP	30	Topilski et al., 2007
Yamaguchi 2003	Prospective	Drug-induced LQTS (10 different drugs)	Bepridil, pirmenol, ranitidine, disopyramide, probucol, aprindine, diazepam, pilsicainide, clonazepam	V5	Tangent method	TdP	27	66	29	TdP	12	Yamaguchi et al., 2003

*LQTS, long QT syndrome; TdP, torsade de pointes*.

**Figure 1:**
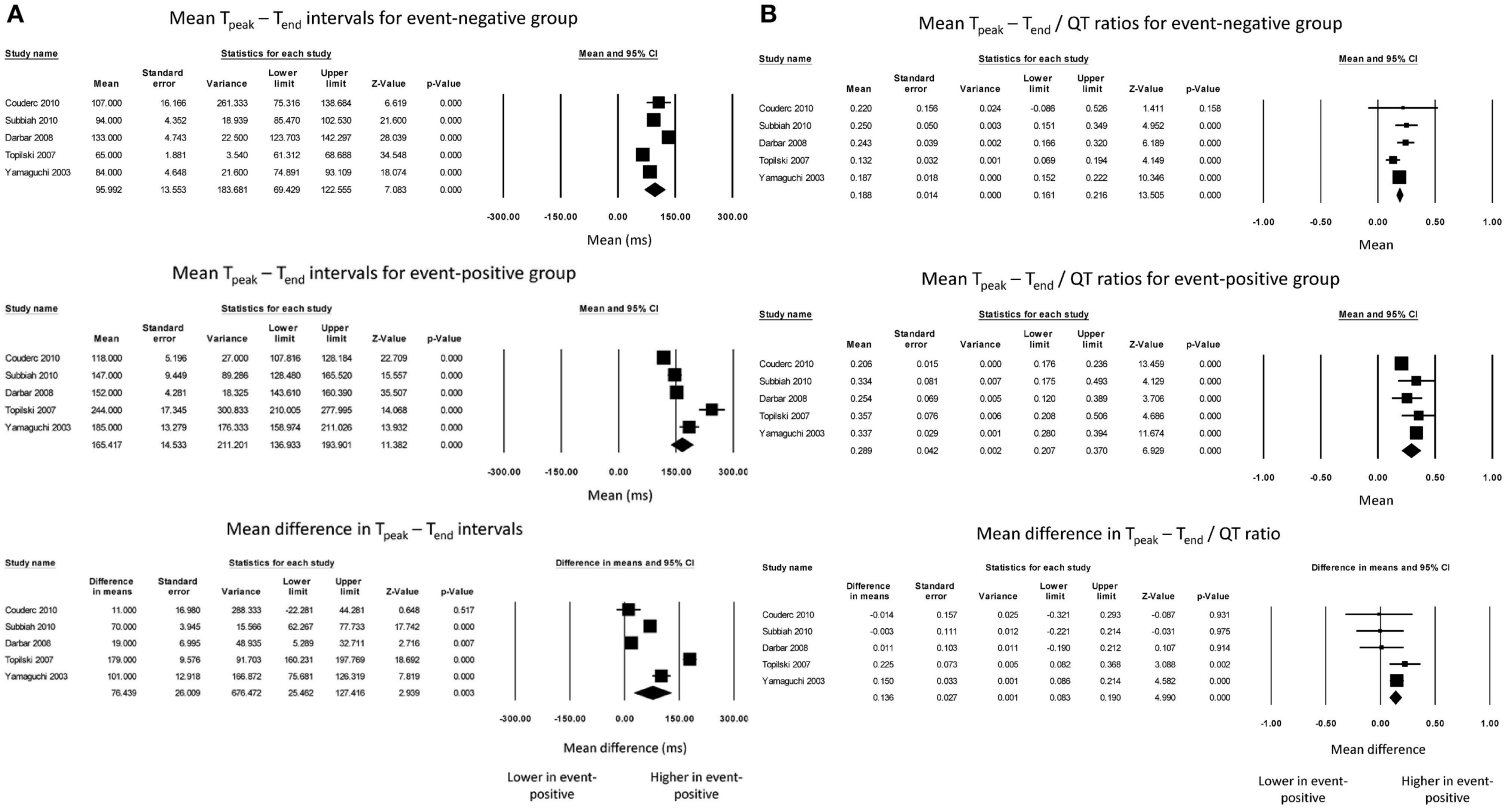
Forest plot demonstrating the **(A)** mean T_peak_ – T_end_ intervals in the event-negative group (top panel), event-positive group (middle panel) and difference between both groups (bottom panel) in acquired QT prolongation, and **(B)** mean T_peak_ – T_end_/QT ratios in the event-negative group (top panel), event-positive group (middle panel) and difference between both groups (bottom panel) in acquired QT prolongation.

The T_peak_ – T_end_ intervals for event-negative (Figure 1A, top panel) and event-positive groups (Figure 1A, middle panel) were 96 ± 14 and 165 ± 15, respectively, with a significant mean difference of 76 ± 26 ms (*P* = 0.003, *I*^2^ = 98%) (Figure 1A, bottom panel). Moreover, the T_peak_ – T_end_/QT ratio for event-negative (Figure 1B, top panel) and event-positive (Figure 1B, middle panel) groups were 0.19 ± 0.01 and 0.29 ± 0.04, respectively, with a significant mean difference of 0.14 ± 0.03 (*P* < 0.0001; *I*^2^ = 29%) (Figure 1B, bottom panel). The results from bias analyses are shown in the Supplementary Material.

**Figure 2:**
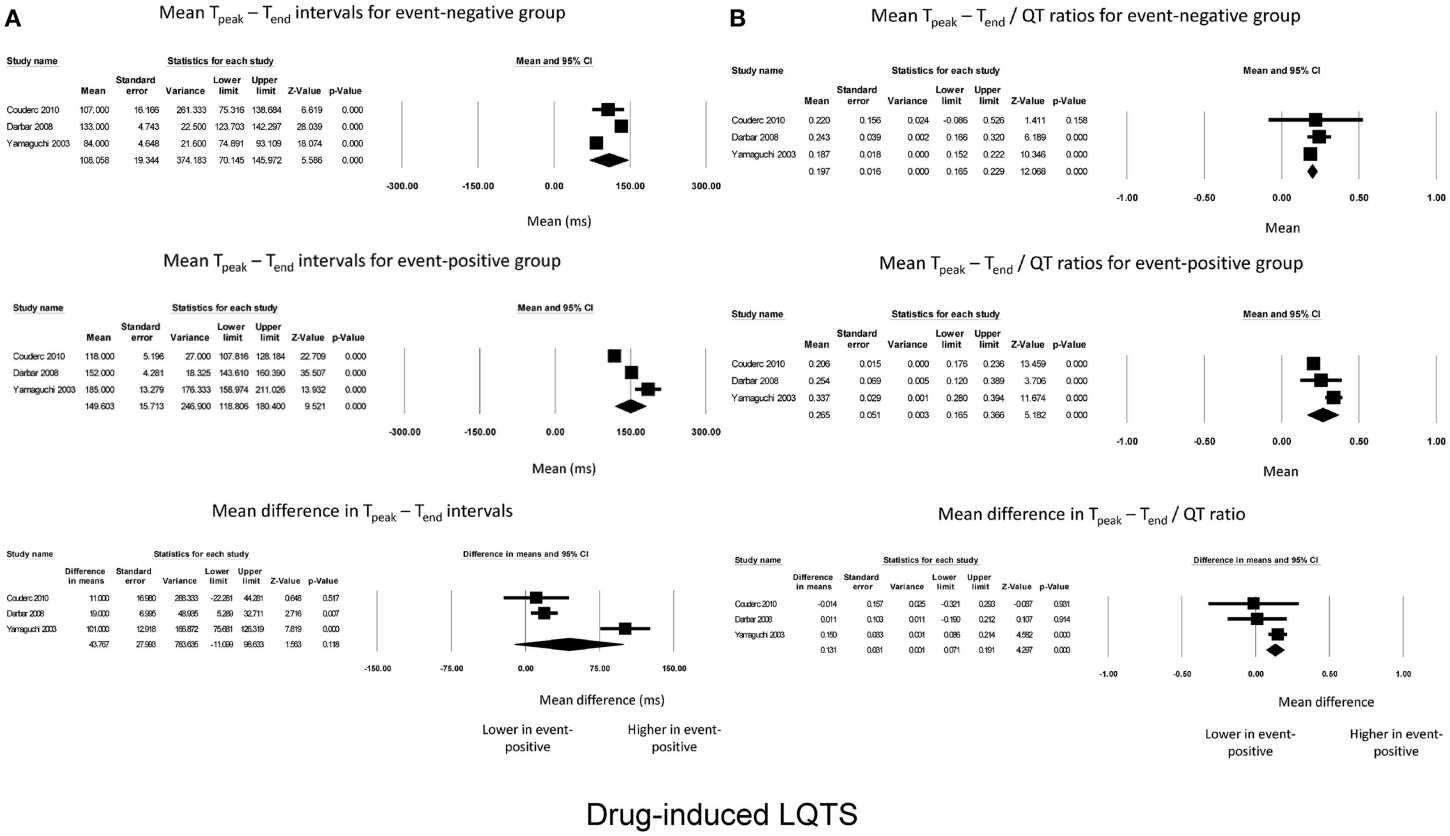
Forest plot demonstrating the **(A)** mean T_peak_ – T_end_ intervals in the event-negative group (top panel), event-positive group (middle panel) and difference between both groups (bottom panel) in acquired QT prolongation, and **(B)** mean T_peak_ – T_end_/QT ratios in the event-negative group (top panel), event-positive group (middle panel) and difference between both groups (bottom panel) in drug-induced QT prolongation.

Subgroup analysis based on the cause of acquired LQTS was performed. A meta-analysis of three studies examining drug-induced LQTS found that T_peak_ – T_end_ intervals for event-negative (Figure 2A, top panel) and event-positive groups (Figure 2A, middle panel) took values of 108 ± 19 and 149 ± 16, respectively. However, the mean difference of 44 ± 28 ms did not reach statistical significance (*P* = 0.12; I^2^ = 94%; Figure 2A, bottom panel). T_peak_ – T_end_ / QT ratios took values of 0.20 ± 0.02 and 0.27 ± 0.05 for event-negative (Figure 2B, top panel) and event-positive (Figure 2B, middle panel) groups, respectively, with a significant mean difference between both groups (0.13 ± 0.03, *P* < 0.0001; I^2^ = 22%; Figure 2B, bottom panel).

**Figure 3:**
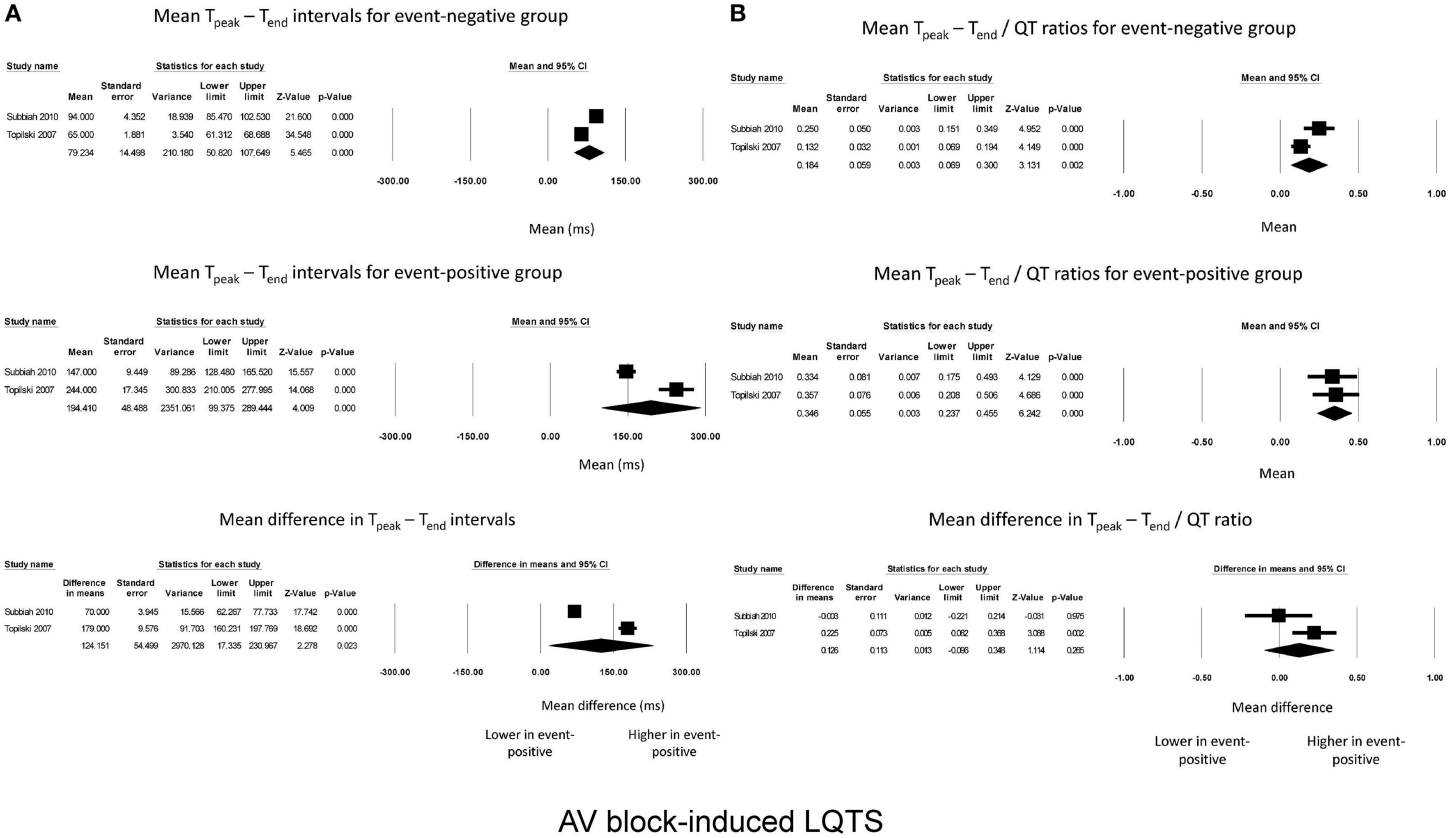
Forest plot demonstrating the **(A)** mean T_peak_ – T_end_ intervals in the event-negative group (top panel), event-positive group (middle panel) and difference between both groups (bottom panel) in acquired QT prolongation, and **(B)** mean T_peak_ – T_end_/QT ratios in the event-negative group (top panel), event-positive group (middle panel) and difference between both groups (bottom panel) in AV block-induced QT prolongation.

For AV block-induced LQTS, two studies were meta-analyzed. T_peak_ – T_end_ intervals for event-negative (Figure 3A, top panel) and event-positive groups (Figure 3A, middle panel) were 79 ± 14 and 194 ± 49, respectively, leading to a significant mean difference of 124 ± 54 ms (*P* = 0.02; I^2^ = 99%; Figure 3A, bottom panel). By contrast, T_peak_ – T_end_ / QT ratios took values of 0.18 ± 0.06 and 0.5 ± 0.06 for event-negative (Figure 3B, top panel) and event-positive (Figure 3B, middle panel) groups, respectively, but the mean difference between both groups was not significant (0.13 ± 0.11, *P* = 0.27; I^2^ = 66%; Figure 3B, bottom panel).

**Figure 4:**
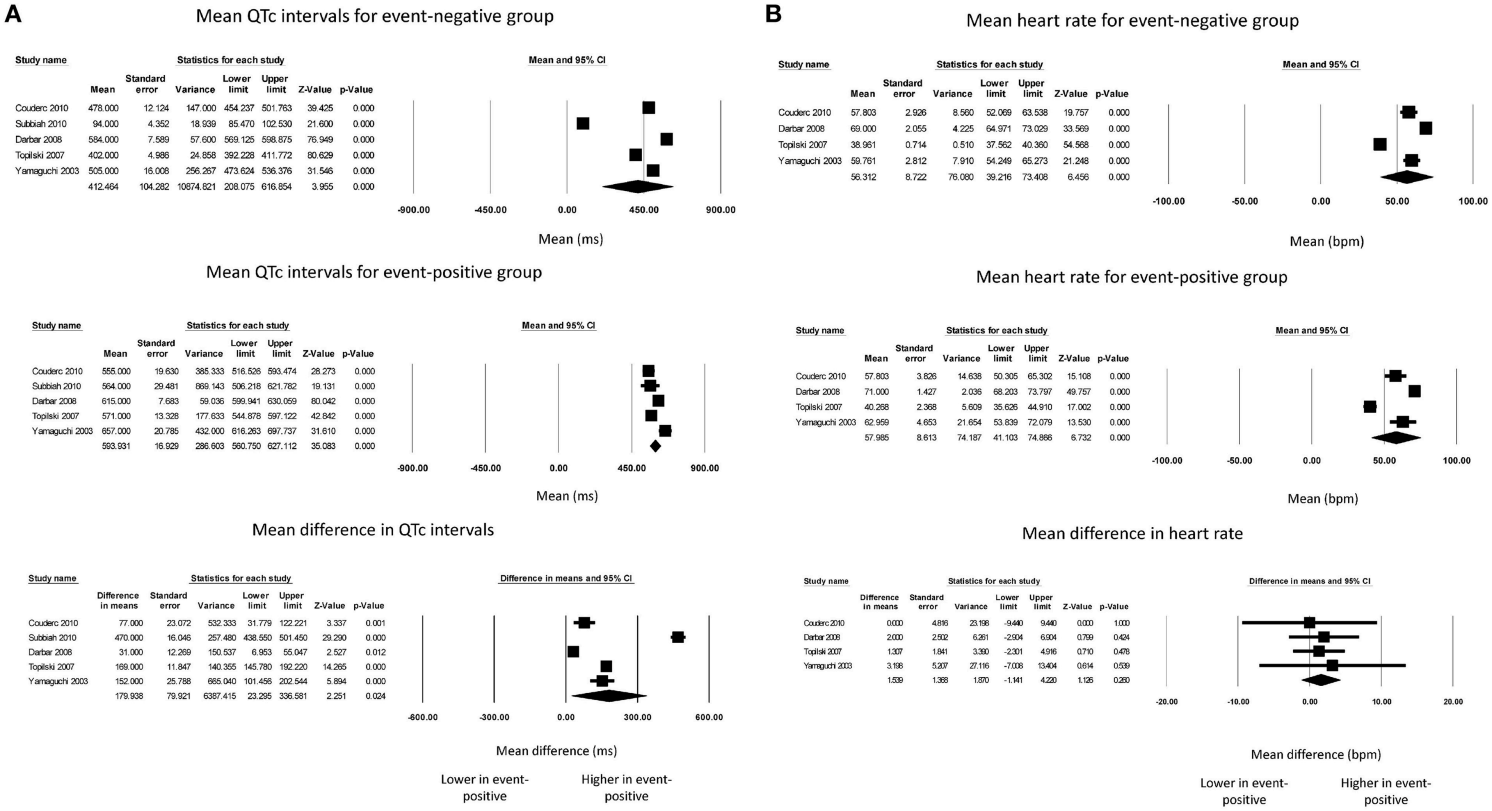
Forest plot demonstrating the **(A)** mean QTc intervals in the event-negative group (top panel), event-positive group (middle panel) and difference between both groups (bottom panel) in acquired QT prolongation, and **(B)** mean heart rate in the event-negative group (top panel), event-positive group (middle panel) and difference between both groups (bottom panel) in acquired QT prolongation.

Finally, QTc intervals for event-negative (Figure 4A, top panel) and event-positive groups (Figure 4A, middle panel) took values of 412 ± 104 and 594 ± 17, respectively, with a significant difference between both groups (180 ± 80 ms; *P* = 0.02; I^2^ = 99%; Figure 4A, bottom panel). Heart rates for event-negative (Figure 4B, top panel) and event-positive groups (Figure 4B, middle panel) took values of 56 ± 9 and 58 ± 9, respectively, with no significant mean difference between both groups (2 ± 1 ms; *P* = 0.26; I^2^ = 0%; Figure 4B, bottom panel).

## Discussion

The major findings of this systematic review and meta-analysis of 308 acquired LQTS subjects are that T_peak_ – T_end_ intervals were longer by 76 ± 26 ms and T_peak_ – T_end_/QT ratios were higher by 0.14 ± 0.03 in those who exhibited adverse events compared with those who did not. QTc were also longer by 180 ± 80 ms but no difference in heart rate was observed.

T_peak_ – T_end_ represent the global dispersion of repolarization (Xia et al., 2005) and its prolongation has been associated with arrhythmogenesis in conditions such as Brugada syndrome, heart failure, myocardial infarction (Tse et al., 2017a,b). Recently, our group conducted a meta-analysis of the predictive values of T_peak_ – T_end_ and T_peak_ – T_end_ / QT ratios in congenital LQTS (Tse et al., 2018). This study extends these findings by providing evidence that T_peak_ – T_end_ intervals and T_peak_ – T_end_/QT ratios are valuable for risk stratification in acquired QT prolongation. Importantly, it shows that such have different risks of adverse outcomes, and that T_peak_ – T_end_ intervals, T_peak_ – T_end_/QT ratios and QTc intervals can be used to identify those who are most at risk. Besides the studies included in this meta-analysis, a large retrospective study compared the outcomes between 293 acquired LQTS subjects and 542 subjects without LQTS (Yu et al., 2017). They found significantly longer QTC intervals associated with higher incidences of life-threatening ventricular arrhythmias as well as one-year mortality in the acquired LQTS group. However, there was no further risk stratification within the acquired LQTS group into survivors and non-survivors using the different ECG indices reported here. Further work is needed to examine whether T_peak_ – T_end_, T_peak_ – T_end_/QT ratio and QTc interval are significant predictors of arrhythmic and mortality events, and to compare their relative sensitivity and specificity.

In our meta-analysis, patients with acquired LQTS due to both drugs and complete AV block were included. Clearly, the mechanisms underlying QT prolongation in these respective groups are different. Thus, drugs that inhibit the outward potassium ion channels can lead to delayed ventricular repolarization, leading to action potential prolongation and lengthening of the QT interval. Whilst drug or metabolic causes of QT lengthening are more common, the original descriptions of *torsade de pointes* associated with QT prolongation by Dessertenne involved complete AV block (Dessertenne, 1966). The underlying causes may be dysfunction of more than one potassium ion channel (Topilski et al., 2007). Our subgroup analysis found that T_peak_ – T_end_/QT ratios, but not T_peak_ – T_end_ intervals, were higher in event-positive subjects with drug-induced LQTS, whereas the converse was true for patients with AV block. However, this may be due to the small sample size and number of studies included.

In addition to QT, QTc, T_peak_ – T_end_ intervals and T_peak_ – T_end_/QT ratio, additional ECG parameters have been proposed for risk stratification (Tse and Yan, 2016). Static markers such as JT_Peak_ prolongation have been shown to predict adverse events in drug-induced LQTS beyond QTc (Johannesen et al., 2016). A new biomarker termed index of Cardiac Electrophysiological Balance (iCEB), an ECG surrogate of excitation wavelength (Tse, 2016), has been shown to predict ventricular arrhythmias associated with dofetilide-induced QT prolongation (Lu et al., 2013) and beyond (Robyns et al., 2016). Dynamic markers include T-wave alternans (TWA) (Zareba et al., 1994), microvolt TWAs (Takasugi et al., 2016), beat-to-beat variability in repolarization (Couderc et al., 2007, 2009; Hinterseer et al., 2008, 2009, 2010; Pueyo et al., 2011) and restitution indices (Nicolson et al., 2012, 2014), all of which have demonstrated predictability for arrhythmic outcomes. These dynamic markers may be provide additional value beyond QTc intervals, as illustrated by the findings that short-term variability in QT intervals was higher in drug-induced LQTS compared to controls, whereas QTc interval was not significantly different between these two groups (Hinterseer et al., 2008). It is unsurprising that variability in repolarization, rather than just the baseline repolarization properties, can further provide risk stratification because arrhythmias do not occur in every individual with prolonged QTc or T_peak_ – T_end_ intervals. Instead, these dynamic factors can either initiate the arrhythmia or provide an additional abnormal substrate that further elevates the risk of re-entry (Thomsen et al., 2004; Tse et al., 2016b).

## Limitations

The following limitations should be recognized. Firstly, the heterogeneity was substantial in our meta-analysis of the mean difference for T_peak_ – T_end_ intervals, with *I*^2^ taking a value of 98%. This compared to a low level of heterogeneity for T_peak_ – T_end_/QT ratios with I^2^ of 29%. The reason may be due to different methods for determining T_end_, which significantly affects the values of T_peak_ – T_end_ intervals obtained. Two studies used the tangent method by taking the intersection of a tangent to the steepest downslope of the dominant repolarization wave with the isoelectric line, whereas the remaining study used the point at which the T-wave intersected with the isoelectric line. By contrast, T_end_ cancels out in T_peak_ – T_end_/QT ratios as it appears in both the numerator and denominator, thereby reducing the influence of T_end_ on the calculated ratio. Secondly, considerable inter-observer variability is likely present because T_peak_ – T_end_ were measured by investigators from different research groups. Thirdly, recall bias may have been present in the retrospective studies, which could have also contributed to some of the heterogeneity observed. Fourthly, the baseline characteristics of the included studies were different, in that one study examined outcomes in the pediatric population whereas two studies were on adult populations. Fifthly, electrolyte abnormalities, most frequently hypokalemia, can also lead to delayed repolarization and consequently QT lengthening (Lee et al., 2003; Hanton et al., 2007; Tse et al., 2016a,c). Thus, potassium levels can be a confounder in further altering the QT interval. Further studies should therefore specify the plasma K+ concentrations at the time of ECG measurements where possible. Finally, although traditionally prolonged QT intervals have been categorized into congenital and acquired causes, recent epidemiological studies reveal a complex relationship between genetics and the environment. Specifically, missense variants in ion channel genes such as the SCN5A that encode for the cardiac voltage-gated sodium channel, can potentiate the effects of hypokalaemia-induced QT prolongation (Akylbekova et al., 2014).

## Conclusions

In the context of acquired QT prolongation, T_peak_ – T_end_ intervals were longer and T_peak_ – T_end_ / QT ratios were higher in patients showing adverse events than in those who did not suffer from these events. These repolarization indices may therefore be useful for risk stratification in this patient population.

## Author contributions

GT: conception of study, data extraction and analysis, data interpretation, manuscript draft, critical revision of manuscript; MG: data extraction and analysis, data interpretation, manuscript draft, critical revision of manuscript; All authors: data interpretation, critical revision of manuscript; WW and TL: study supervision.

### Conflict of interest statement

The authors declare that the research was conducted in the absence of any commercial or financial relationships that could be construed as a potential conflict of interest.
