# Supplementary material for: Predictive Value of Tpeak – Tend Indices for Adverse Outcomes in Acquired QT Prolongation: A Meta-Analysis
Source: Front Physiol. 2018 Sep 3;9:1226. doi: 10.3389/fphys.2018.01226 (PMC6129769; doi:10.3389/fphys.2018.01226)
Supplement: Supplementary file 1 [file Data_Sheet_1.PDF]

Quality assessment of included studies using the Newcastle–Ottawa Quality Assessment Scale (NOS)

The point score system evaluated the categories of study participant selection, comparability of the results, and quality of the outcomes. The following characteristics were assessed: a) representativeness of the exposed cohort; b) selection of the non-exposed cohort; c) ascertainment of exposure; d) demonstration that outcome of interest was not present at the start of study; e) comparability of cohorts based on study design or analysis; f) assessment of outcomes; g) follow-up periods that were sufficiently long for outcomes to occur; and h) adequacy of follow-up of cohorts. This scale varied from zero to nine stars, which indicated that studies were graded as poor quality if the score was <5, fair if the score was 5 to 7, and good if the score was >8. Studies with a score equal to or higher than six were included.

Studies	Selection				Comparability	Exposure			Total score (0-9)
	Adequate definition of cases	Representativeness of cases	Selection of controls	Definition of controls		Ascertainment of exposure	Same method of ascertainment for subjects	Non-response rate	
Couderc 2010	*	*	*	*	0	*	*	*	7
Darbar 2008	*	*	*	*	* (age)	*	*	*	8
Topilski 2007	*	*	*	*	* (age)	*	*	*	8
Yamaguchi 2003	*	*	*	*	0	*	*	*	7

Supplementary Table 1. NOS risk of bias scale for case-control studies.

Studies	Selection				Comparability	Outcome			Total score (0-9)
	Representativeness of the exposed Cohort	Selection of the non-exposed cohort	Ascertainment of exposure	Outcome of interest not present at start of study		Assessment of outcome	Adequacy of duration of follow-up	Adequacy of completeness of follow-up	
Subbiah 2010	*	*	*	*	* (age)	*	*	*	8

Supplementary Table 2. NOS risk of bias scale cohort studies.

Bias analysis

Regarding $T_{peak} - T_{end}$ intervals, The Cochran's Q value was greater than the degrees of freedom (6 vs. 4), indicating that the true effect size was different between studies. I^2 took a value of 34%, suggesting a low level of heterogeneity. A funnel plot plotting standard errors against differences in means is shown in **Supplementary Figure 1**. Begg and Mazumdar rank correlation analysis demonstrated that Kendall's Tau took a value of 0.2 with P = 0.62, which suggests no significant publication bias. Egger's test demonstrated no significant asymmetry (intercept 1.5, t-value 1.2; P = 0.32). To identify the source of the heterogeneity, sensitivity analysis was performed by removing one study at a time. However, this did not significantly influence the mean difference (**Supplementary Figure 2**), suggesting that no single study was responsible for the heterogeneity observed in this meta-analysis.

For $T_{peak} - T_{end}$ / QT ratio**,** The Cochran's Q value was less than the degrees of freedom (0.2 vs. 3), indicating that the true effect size was not significantly different between the included studies. I^2 took a value of 0%, suggesting no heterogeneity. A funnel plot plotting standard errors against differences in means is shown in **Supplementary Figure 3**. Begg and Mazumdar rank correlation analysis demonstrated that Kendall's Tau took a value of 0 with P = 1.00, which suggests no publication bias. Egger's test demonstrated no significant asymmetry (intercept 0.1, t-value 0.3; P = 0.81). To identify the source of the heterogeneity, sensitivity analysis was performed by removing one study at a time to calculate the pooled OR. However, this did not significantly influence the mean difference (**Supplementary Figure 4**), suggesting that no single study was responsible for the heterogeneity observed in this meta-analysis.

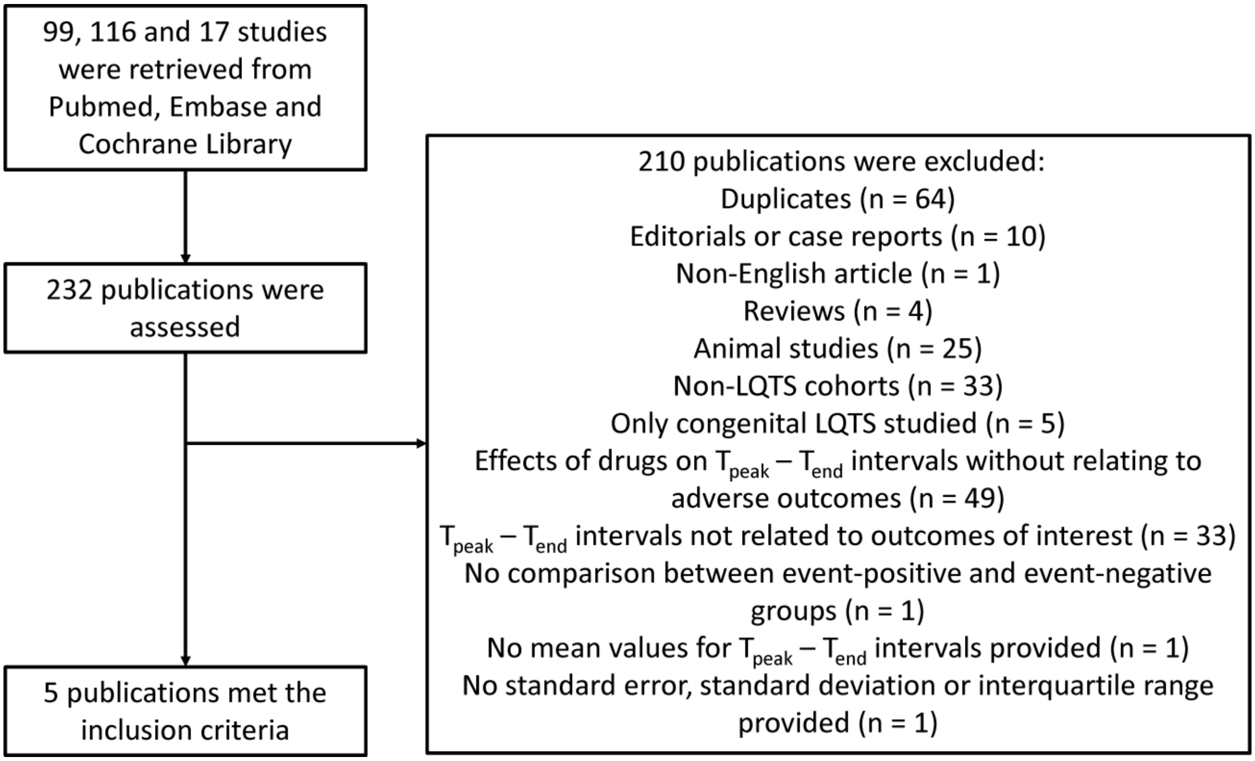

Supplementary Figure 1. Flow diagram of the study selection process.

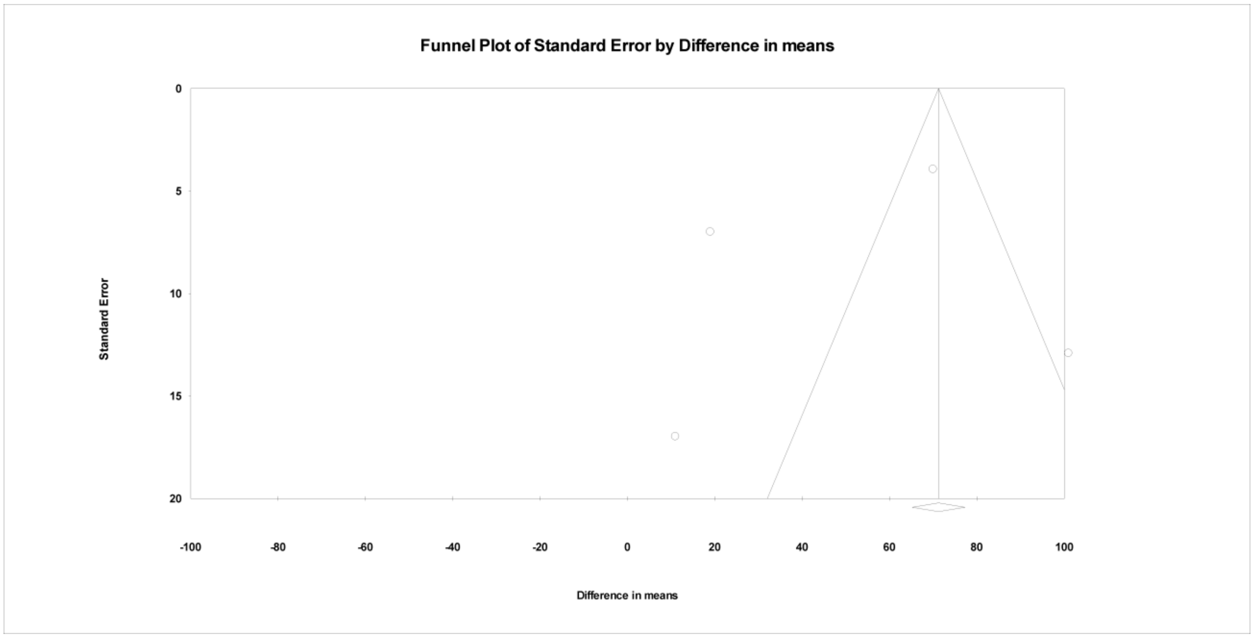

Supplementary Figure 2. Funnel plot of standard errors against differences in means for T_{peak} – T_{end} interval.

Mean difference in T_{peak} – T_{end} (sensitivity analysis)

Study name	Point	Standard error	Variance	Lower limit	Upper limit	Z-Value	p-Value
	Statistics with study removed						
Couderc 2010	91.846	29.213	853.394	34.590	149.103	3.144	0.002
Subbiah 2010	77.783	43.572	1898.489	-7.616	163.182	1.785	0.074
Darbar 2008	91.063	31.325	981.279	29.666	152.460	2.907	0.004
Topilski 2007	50.891	18.419	339.259	14.790	86.991	2.763	0.006
Yamaguchi 2003	70.333	30.849	951.680	9.870	130.797	2.280	0.023
	76.439	26.009	676.472	25.462	127.416	2.939	0.003

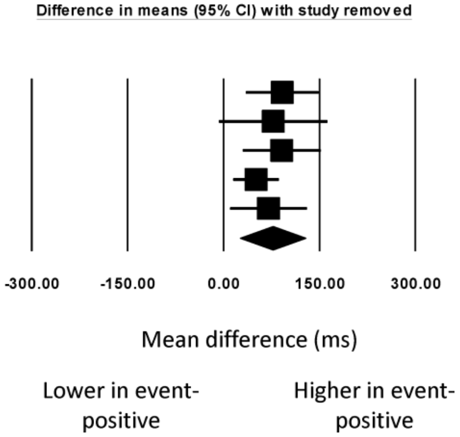

Supplementary Figure 3. Forest plot demonstrating the results of sensitivity analysis by removing one study at a time for mean differences of T_{peak} – T_{end} intervals.

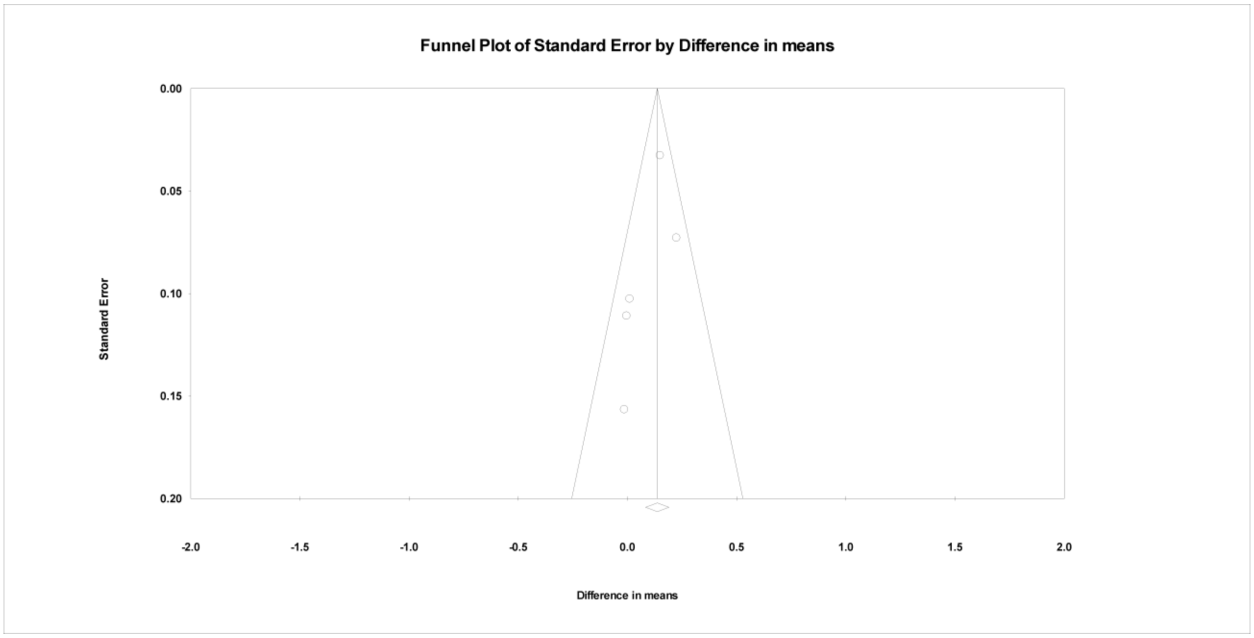

Supplementary Figure 4. Funnel plot of standard errors against differences in means for T_{peak} – T_{end} / QT ratio.

Mean difference in T_{peak} – T_{end} / QT ratio (sensitivity analysis)

Study name	Statistics with study removed						
	Point	Standard error	Variance	Lower limit	Upper limit	Z-Value	p-Value
Couderc 2010	0.141	0.028	0.001	0.087	0.196	5.084	0.000
Subbiah 2010	0.145	0.028	0.001	0.090	0.201	5.157	0.000
Darbar 2008	0.146	0.028	0.001	0.090	0.202	5.148	0.000
Topilski 2007	0.122	0.029	0.001	0.064	0.180	4.134	0.000
Yamaguchi 2003	0.105	0.050	0.002	0.008	0.203	2.116	0.034
	0.136	0.027	0.001	0.083	0.190	4.990	0.000

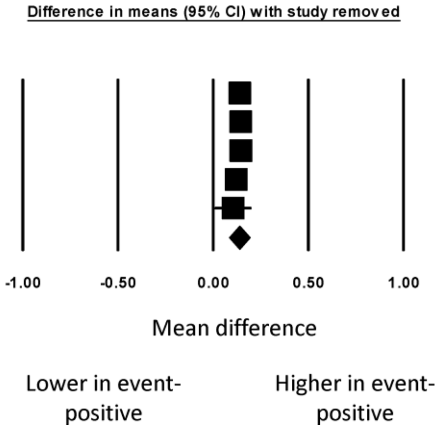

Supplementary Figure 5. Forest plot demonstrating the results of sensitivity analysis by removing one study at a time for mean differences of T_{peak} – T_{end} /QT ratio.
